# Congestion Control and Traffic Differentiation for Heterogeneous 6TiSCH Networks in IIoT

**DOI:** 10.3390/s20123508

**Published:** 2020-06-21

**Authors:** Hossam Farag, Patrik Österberg, Mikael Gidlund

**Affiliations:** Department of Information Systems and Technology, Mid Sweden University, 851 70 Sundsvall, Sweden; patrik.osterberg@miun.se (P.Ö.); mikael.gidlund@miun.se (M.G.)

**Keywords:** Industrial IoT, 6TiSCH, RPL, trickle timer, priority, congestion, traffic differentiation

## Abstract

The Routing Protocol for Low power and lossy networks (RPL) has been introduced as the de-facto routing protocol for the Industrial Internet of Things (IIoT). In heavy load scenarios, particular parent nodes are likely prone to congestion, which in turn degrades the network performance, in terms of packet delivery and delay. Moreover, there is no explicit strategy in RPL to prioritize the transmission of different traffic types in heterogeneous 6TiSCH networks, each according to its criticality. In this paper, we address the aforementioned issues by introducing a congestion control and service differentiation strategies to support heterogeneous 6TiSCH networks in IIoT applications. First, we introduce a congestion control mechanism to achieve load balancing under heavy traffic scenarios. The congestion is detected through monitoring and sharing the status of the queue backlog among neighbor nodes. We define a new routing metric that considers the queue occupancy when selecting the new parent node in congestion situations. In addition, we design a multi-queue model to provide prioritized data transmission for critical data over the non-critical ones. Each traffic type is placed in a separate queue and scheduled for transmission based on the assigned queue priority, where critical data are always transmitted first. The performance of the proposed work is evaluated through extensive simulations and compared with existing work to demonstrate its effectiveness. The results show that our proposal achieves improved packet delivery and low queue losses under heavy load scenarios, as well as improved delay performance of critical traffic.

## 1. Introduction

The Industrial Internet of Things (IIoT) is the sub-category of the IoT that targets the industrial sector to improve productivity and efficiency [1]. Unlike consumer IoT, IIoT applications are characterized by stringent communication requirements in terms of reliability and delay [2]. The IPv6 over Time-Slotted Channel Hopping (6TiSCH) working group was established in 2013 with the aim to enable industrial-grade IPv6 networks to foster IIoT [3]. The 6TiSCH network is a key component to enable the adoption of IPv6 in industrial standards and the convergence of Operational Technology (OT) with Information Technology (IT) where industrial devices (e.g., sensors, actuators, robots, etc.) are enabled to connect to the cloud. The collected information from the industrial site is integrated via a control and management platform to improve the operational efficiency and productivity of the manufacturing process [4]. In this context, the 6TiSCH working group is developing solutions for missing components, such as schedule management, deterministic IPv6 flows, link management, and routing [5].

6TiSCH networks are Low-power and Lossy Networks (LLNs) that are constructed using low-cost and resource-constrained devices, namely LLN devices [6,7]. In 6TiSCH networks, communication routes are constructed and maintained through the Routing Protocol for LLN (RPL) [6]. RPL organizes the 6TiSCH network as a Destination-Oriented Acyclic Graph (DODAG) rooted at the sink node, namely the DODAG root. Each node is attached to a parent node that relays all its sensory information to the DODAG root. RPL employs the concept of RANKto define the relative position of a node with respect to the DODAG root. In RPL, the Objective Function (OF) describes the rules to compute the RANK and how it should be used to select the preferred parent [7]. Currently, there are two OFs defined by the RPL standard, OF zero (OF0) [8] and Minimum Rank with Hysteresis OF (MRHOF) [9]; however, there is no obligation to use a specific OF. Information about the RANK and OF is advertised through DODAG Information Object (DIO) messages, whose transmission interval is controlled by the Trickle timer algorithm [10]. Based on the information received by the DIO message and the adopted OF, the node selects a preferred parent from its candidate parent set. In heavy traffic load circumstances, parent nodes are likely to be congested due to the increased forwarding rate and the limited buffer size of LLN nodes. In the RPL standard, a parent node is selected either based on hop-counts or the link quality [6] without considering the congestion level causing an imbalanced network. Congestion has a profound impact on the network performance in terms of packet loss, delay, and energy consumption, and experimental measurements revealed that in high traffic scenarios, the main cause of packet loss is due to congestion [11]. The current RPL specifications do not specify how to detect and control congestion in 6TiSCH networks.

Moreover, in many industrial applications, such as monitoring and control scenarios, sensor nodes generate different traffic types with varying real-time requirements [12]. In certain scenarios, e.g., emergencies, critical traffic has higher importance than other types of traffic and must be delivered within a limited time to maintain stability, functionality, and to avoid dangerous situations. For data transmission scheduling, the current version of RPL considers the nodes to utilize either the First-In First-Out (FIFO) policy [13] or Last-In First-Out (LIFO) policy [14] in their output buffer. RPL has no explicit mechanism to efficiently handle the transmission of heterogeneous traffic based on the corresponding criticality and performance requirements. Accordingly, critical packets may be blocked and transmitted after non-critical ones, hence violating their timing limits. Therefore, traffic priority and network congestion are key research challenges in heterogeneous 6TiSCH networks in IIoT applications.

This paper presents a congestion control and service differentiation framework to support heterogeneous 6TiSCH networks in IIoT applications. Principally, our proposed approach investigates two key research questions: first, how to achieve fair load distribution in imbalanced 6TiSCH networks and achieve improved packet delivery performance in heavy traffic scenarios; second, how to guarantee prioritized and low delay transmissions of critical data over the non-critical in heterogeneous 6TiSCH networks. To this end, the first question is addressed through a congestion detection and control mechanism, while the second question is addressed through a multi-queue model, both combined in the proposed congestion control and service differentiation framework to achieve load balancing and improved packet delivery for all types of traffic and enhanced delay performance for critical data. To the best of our knowledge, this is the first work to address congestion and traffic differentiation issues in heterogeneous 6TiSCH networks. The key contributions of our proposed work can be summarized as follows

- We introduce a congestion control approach to achieve load balancing and improve network performance in terms of packet delivery under heavy load conditions. In the proposed approach, a new joint routing metric is defined to select parent nodes considering queue occupancy along with the hop distance and link quality metrics.
- To further support the functionality of the above strategy, we propose a Trickle timer reset strategy to detect overloaded nodes and to react to congestion in a timely fashion while maintaining minimum network overhead.
- Moreover, we design a multi-queue model where each node uses three different queues corresponding to three traffic categories, which is the typical case in most IIoT scenarios. Each queue is given a transmission priority where packets from higher priority queues are transmitted first. In addition, we provide a stochastic mathematical model to formulate the average queue waiting time of the proposed multi-queue model.
- We evaluate the performance of the proposed work through extensive discrete-time simulations and conduct performance comparisons with existing work to demonstrate its effectiveness. The results show that our proposed framework achieves improved packet delivery and low queue losses under heavy load scenarios, as well as improved real-time performance of critical traffic.

The remainder of this paper is organized as follows. Section 2 discusses the problem statement and related work. The proposed congestion control method is introduced in Section 3. Section 4 presents the multi-queue model and its corresponding mathematical analysis. Performance evaluations are given in Section 5, followed by the conclusion and future work in Section 6.

## 2. Problem Statement and Related Work

In this section, we first highlight the congestion and traffic priority issues in RPL networks, then we discuss related works in this context.

### 2.1. Problem Statement

Although RPL has been designed to meet the requirements of LLNs, there are issues still challenging to satisfy the stringent requirements of heterogeneous 6TiSCH networks in IIoT applications. This paper mainly tackles two issues.

The first issue is the congestion control under heavy traffic load and imbalanced DODAG construction. RPL is mainly designed to handle traffic in LLNs under light traffic conditions. However, in high traffic conditions, parent nodes are prone to congestion problems, especially those close to the DODAG root. In RPL, the considered OF allows each node to select its preferred parent based on the hop-count and link quality, i.e., Expected Transmission Count (ETX ), regardless of the queue occupancy of neighboring nodes. Hence, the OF does not reflect the congestion of parent nodes, which in turn degrades network performance in terms of packet loss and delay. The problem even exists under light traffic condition where there is unfair load distribution among parent nodes.

**Figure 1:**
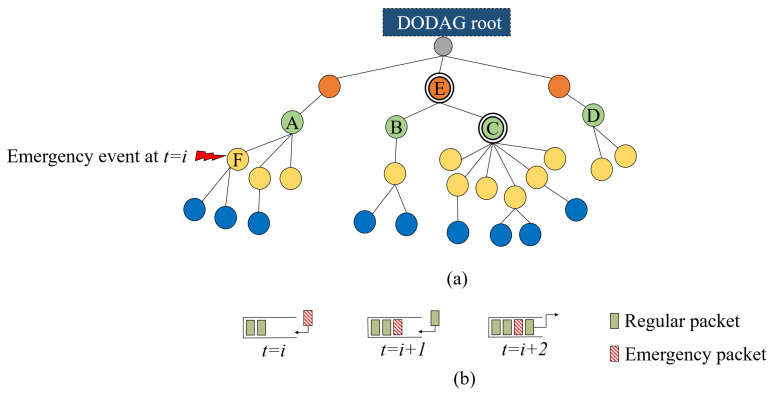
6TiSCH network scenario: (**a**) routing topology; (**b**) LIFO queue model of node *F*.

**Figure 2:**
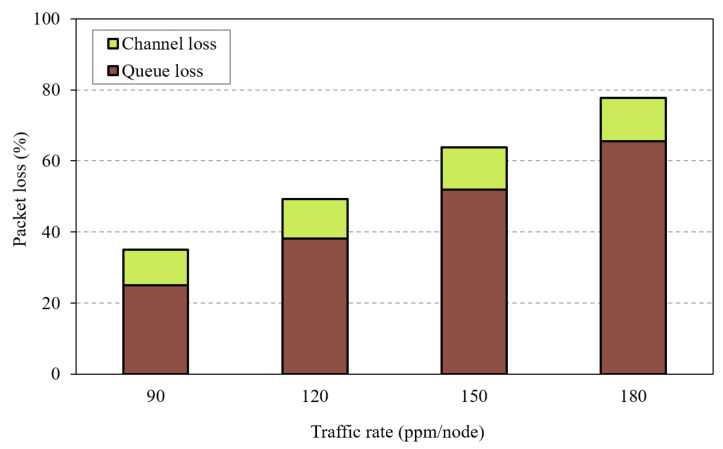
Packet loss for different traffic rates.

To better understand the problem, consider the routing topology shown in Figure 1a. Node *C* is overloaded with children compared to nodes *A*, *B*, and *D*, which have the same RANK. This in turn incurs a significant traffic load at node *E*, the parent node of node *C*, which is responsible for forwarding the traffic of more than 50% of the network. In high traffic conditions, the children of *C* send packets with high rates, which leads to buffer overflow at node *C*, while nodes *A*, *B*, and *D* maintain a stable buffer status. The same, and even worse, occurs at node *E*. Typically, LLN devices are resource-constrained and have small queue sizes. These small queues start to overflow before the congestion becomes heavy enough to be detected through the ETX and Trickle timer. The children of *C* are not aware of the congestion problem, and hence, they continue to transmit packets to *C* as they measure low ETX from their perspective. Moreover, the Trickle timer is not aware of the congestion situation; thus, the node cannot change its congested parent in a timely manner. Therefore, the conventional Trickle timer and using ETX for parent selection is not reasonable for load balancing in RPL-based networks. In Figure 2, we investigate packet losses in an RPL network with OF0 under different traffic load conditions. From this figure, we can clearly note that queue losses constitute the dominant part of packet losses compared to channel losses (almost 16%) even in high traffic load conditions. This in turn will be misleading for OF0 to change the parent when the node is congested, thus the need for an efficient parent selection and congestion control mechanism.

The second issue is the traffic priority in heterogeneous 6TiSCH networks. RPL defines no mechanism to prioritize the transmission of heterogeneous traffic types in 6TiSCH networks according to their timing requirements. All packets are placed in a single queue and scheduled either in an LIFO or FIFO fashion. High priority data are mainly characterized by tight timing constraints, and if they arrive too late, they are of limited use and could lead to system failure, production loss, or even dangerous situations [12]. Both queue scheduling policies adopted by the RPL may cause higher priority transmissions to violate its timing limits when blocked by the transmission of lower priority ones, which typically have relaxed timing requirements. Therefore, the queue scheduling of the current RPL version is an inefficient solution in IIoT applications with heterogeneous traffic. To further illustrate the problem, consider the 6TiSCH network scenario depicted by Figure 1a. As a result of a particular emergency event, an emergency packet arrives at the output queue of node *F* at time t=i, as shown in Figure 1b. Due to its criticality, this packet should be transmitted first with the highest priority. At t=i+1, a regular packet joins the output queue of the same node that is either coming from one of its children or generated by node *F* itself. Considering the LIFO policy for this case, at t=i+2, the emergency packet transmission is blocked due to the transmission of the recently arrived regular packet, which may cause the emergency packet to miss its deadline. A similar situation would happen if we consider the FIFO policy. Therefore, for such applications, a proper packet scheduling method is needed.

### 2.2. Related Work

In recent years, several works were introduced to improve the performance of RPL-based networks in terms of different metrics [14,15,16]. However, most of these efforts overlook reliability and real-time aspects, which are crucial for IIoT applications especially when involving transmission of heterogeneous data. Routing and data transmission scheduling are the key components that directly affect data transport capabilities in terms of reliability and real-time delivery. In the context of data transmission scheduling, a number of research efforts were proposed to support the real-time transmission of critical data in industrial applications [12,17,18,19]. These works are mainly based on improved channel access mechanisms that allow critical data to gain higher priority to access the channel over the non-critical ones. However, those works are only applicable for single-hop networks. However, it has been shown that the multi-queuing strategy plays a major role in affecting the Quality-of-Service (QoS) requirements of wireless sensor networks with multiple traffic types [20]. The work in [21] introduced EARS, an emergency packet scheduling scheme for IoT in smart cities. In EARS, each incoming packet is placed in the corresponding queue based on its priority and deadline information where emergency packets are always processed and transmitted first. A dynamic multilevel priority packet scheduling scheme was proposed in [22], where each node had three levels of priority queues. In this approach, real-time/emergency packets are placed in the highest priority queue and can preempt other queues, while non-real time data are placed into the other two queues and processed based on the shortest job first scheduler. Another multilevel queuing approach was proposed in [23] where each node utilizes a number of queues based on its location. The node decides the packet priority based on its hop count, and accordingly, the packet is placed in the relevant queue. In [24], the authors proposed a cloud-assisted priority-based scheme. The prioritized data packets received by each cluster head are sent to one of two queues: the high priority queue or the low priority queue where the preemptive M/G/1queuing model is employed. All the aforementioned multi-queuing approaches have been shown to perform well in light traffic conditions; however, the negative impact of congestion in heavy traffic scenarios in 6TiSCH networks is not considered, which has a direct effect on the reliability real-time communications of high priority traffic.

Several works have been introduced to control the congestion in RPL-based networks. The MLEqprotocol [25] was proposed to handle the congestion problem using multiple gateways in IPv6 over Low-Power Wireless Personal Area Networks (6LoWPAN). Congested gateways share their load in a distributed fashion to achieve a global load fairness; however, the approach is not applicable in RPL networks with a single gateway. The works in [26,27,28] proposed to formulate the congestion issue as an optimization problem based on a strictly concave and continuously differentiable function, e.g., congestion cost function or a utility function, where the objective is to select the optimal sending rate to minimize the congestion level. However, this approach is not applicable in many IIoT applications where setting a predefined sending rate is crucial to maintain the network functionality and stability. The authors in [29] proposed M-RPL, a Multi-path extension of RPL to alleviate the congestion problem by providing temporary multiple paths for the congested nodes. Although M-RPL manages to improve the network performance in terms of energy consumption and throughput, it suffers from increased delay when constructing the multi-path routes. Moreover, the conventional Trickle timer utilized by the aforementioned approaches could fail to detect and react to congestion in a timely manner. The authors in [30] proposed CoAR, a Congestion-Aware Routing protocol, where the Trickle timer is reset to its minimum value when a congestion is detected. Although CoAR improves the network performance in congestion situations, the utilized reset strategy increases the network overhead and the energy consumption in turn. The authors in [31] proposed a heuristic algorithm that calculates the redundancy constant of the Trickle timer as a function of the number of neighbor nodes in its vicinity. However, the work overlooked the impact of the proposed algorithm on the QoS metrics of the constructed routes. The Trickle-Dalgorithm was proposed in [32], in which the redundancy constant is adapted using Jain’s index to achieve fairness among nodes while keeping low overhead. According to the obtained results, Trickle-D achieves improved performance in terms of fairness and energy consumption, while other vital metrics such as packet delivery ratio and delay are not considered. Moreover, none of the above works considered the prioritized transmission of critical traffic in heterogeneous 6TiSCH networks.

## 3. The Proposed Congestion Control Mechanism

In this section, we describe the congestion control framework used to achieve load balancing in 6TiSCH networks. We first present a congestion detection and control method, then we describe a mechanism to distribute congestion information between neighbor nodes. Finally, we introduce the proposed Trickle timer reset strategy.

### 3.1. Detecting and Controlling Congestion

Initially, the DODAG is constructed through exchanging the DIO messages between neighbor nodes. First, a node $n_{i}$ generates its parent candidate set $Parent(n_{i})$ as a subset of its neighbor candidate set $N(n_{i})$ as follows:

(1) $$Parent\left(n_{i}\right)=\left\{n_{j}\in N\left(n_{i}\right)|H\left(n_{j}\right)<H\left(n_{i}\right),ETX\left(n_{i},n_{j}\right)<\gamma \right\},$$

where $H(n_{j})$ denotes the hop-count between $n_{j}$ and the DODAG root, $ETX(n_{i},n_{j})$ is the estimated ETX value between $n_{i}$ and $n_{j}$, and γ is a threshold to eliminate neighbors with bad link quality. $ETX(n_{i},n_{j})$ is obtained as [33]:

(2) $$ETX\left(n_{i},n_{j}\right)=\frac{\#\;\mathrm{of}\;\mathrm{total}\;\mathrm{transmissions}\;\mathrm{from}\;n_{i}\;\mathrm{to}\;\;n_{j}}{\#\;\mathrm{of}\;\mathrm{successful}\;\mathrm{transmissions}\;\mathrm{from}\;n_{i}\;\mathrm{to}\;\;n_{j}}.$$

Each node $n_{i}$ selects a preferred parent node $P_{i}$ from its $Parent(n_{i})$ for data forwarding. We consider that the initial $P_{i}$ is selected as the one that has the minimum hop-distance towards the DODAG root [8].

A node $n_{i}$ selects a new parent $P_{i}^{*}$ when changes occur to its $Parent(n_{i})$. In our proposed method, the new parent selection process is triggered if one of the following two criteria are satisfied: the joint Hop-distance and Link quality (HL)-criterion, and the Load-Balancing (LB)-criterion. Based on these criteria, we also introduce two distinct parent selection mechanisms.

#### 3.1.1. The HL-Criterion

The first selection method corresponds to satisfying only the HL-criterion. This case represents light traffic scenarios and mainly aims to select $P_{i}^{*}$ based on link quality and hop-count. The HL-criterion is defined as:

(3) $$R^{HL}\left(P_{i}\right)-R^{HL}\left(P_{i}^{*}\right)>\theta ,$$

where $R^{HL}\left(P_{i}\right)$ is the routing metric with respect to $P_{i}$ and θ is the hysteresis value used to avoid excessive parent switching due to small changes in the routing metric. The HL-criterion is based on the routing metric $R^{HL}\left(p_{i}\right)$ that reflects the link quality and the hop distance for each parent candidate $p_{i}\in Parent\left(n_{i}\right)$. $R^{HL}\left(p_{i}\right)$ is given as:

(4) $$R^{HL}\left(p_{i}\right)=\underset{RANK(p_{i})}{\underset{︸}{H(p_{i})+1}}+ETX\left(n_{i},p_{i}\right).$$

When the HL-criterion in Equation (3) is satisfied, a new parent $P_{i}^{*}$ is selected as:

(5) $$P_{i}^{*}=\min_{p_{i}\in Parent\left(n_{i}\right)}\left\{R^{HL}\left(p_{i}\right)\right\}.$$

Hence, in light traffic conditions, a node selects its new parent mainly based on the link quality and the hop-distance.

#### 3.1.2. The LB-Criterion

As mentioned earlier, the ETX metric is insufficient for the detection of congestion in heavy traffic conditions. The small queues of the LLN nodes start to overflow before the congestion is heavy enough to degrade the ETX and be detected through the HL-criterion. In this case, $n_{i}$ keeps transmitting to its $P_{i}$ even if it suffers from consecutive queue losses.

We introduce the LB-criterion to detect the congestion in parent nodes and change to $P_{i}^{*}$ based on the queue occupancy information to achieve load balancing. The formulation of the LB-criterion is mainly based on the backlog factor $BF(n_{i})$ that represents the queue occupancy of $n_{i}$. $BF(n_{i})$ is defined as the ratio of the number of backlogged packets in the output queue $Q(n_{i})$ to the total queue size $L(n_{i})$. How to set the LB-criterion and $BF(n_{i})$ properly is illustrated as follows.

When $P_{i}$ is congested, $BF(n_{i})$ may be much smaller than $BF(P_{i})$ even if $P_{i}$ suffers from queue losses; hence, $BF(n_{i})$ cannot properly reflect congestion in this case. Furthermore, when $BF(n_{i})$ is high, changing the parent of $n_{i}$ would not help in load balancing, but instead, the children of $n_{i}$ should migrate to another parent to reduce the load on $n_{i}$. $BF(P_{i})$ is also not a proper indicator of congestion, as when the network is balanced, each node will have low $BF(P_{i})$; hence, the LB-criterion would not be satisfied, and parent selection would only be triggered based on the HL-criterion, causing an imbalanced network again. Therefore, we define the LB-criterion as:

(6) $$\max\left\{BF_{max},BF_{max}^{\left[m+1\right]}\left(n_{i}\right)\right\}>\delta ,$$

where $BF_{max}$ is the maximum backlog factor recognized for all parent candidates of $n_{i}$ within the last *m* consecutive slotframes, $BF_{max}^{\left[m+1\right]}\left(n_{i}\right)$ is the maximum backlog factor of all parents of $n_{i}$ in the current slotframe, and δ is the congestion threshold. To further illustrate, the maximum backlog factor of parent nodes recorded by node $n_{i}$ within the $j^{\mathrm{th}}$ slotframe is given as:

$$BF_{max}^{\left[j\right]}\left(n_{i}\right)=\max_{p_{i}\in Parent\left(n_{i}\right)}\left\{BF(p_{i})\right\}.$$

Then, $n_{i}$ maintains these values and calculates the maximum for the recent *m* slotframes as follows:

$$BF_{max}=\max_{j\in \{1,2,...,m\}}\left\{BF_{max}^{\left[j\right]}\left(n_{i}\right)\right\}.$$

Finally, $n_{i}$ uses the sliding window to update $BF_{max}$ for every slotframe, select the maximum of these values, and compare it with the congestion threshold δ as given in (6).

The value of the number of consecutive slotframes *m* in (6) is a configurable parameter and is empirically selected considering the following conditions regarding the selection of its lower and upper limits. First, *m* is selected such that $\left(m\times T\right)>I_{min}$, where *T* is the duration of a slotframe and $I_{min}$ is the minimum interval of the Trickle timer. This is because the value of $BF_{max}^{\left[j\right]}\left(n_{i}\right)$ of a node $n_{i}$ is determined using the backlog information of its parents $BF(p_{i})$ according to (6), and such information is propagated through the DIO messages whose interval is determined through the Trickle timer, i.e., collecting the information at least every $I_{min}$. Moreover, the node needs to maintain a reasonable record of past congestion events in order to be more aware of the congestion situation of its parents and avoid hasty parent changes. Second, LLN nodes are resource-constrained devices that typically have limited storage capacity, and increasing *m* means storing more values of $BF(p_{i})$, which might be a limitation to the node if it increases beyond a certain value. Therefore, the upper bound of *m* is mainly based on the available resources. Furthermore, it is pointless to increase *m* to store too old history of $BF(p_{i})$.

The threshold value 0<δ<1 determines when to perform parent change as a result of congestion. Empirically, we consider δ=0.5, which means that a new parent $P_{i}^{*}$ should be selected when the congestion level is above 50%. A higher value of δ could cause a delayed detection of congestion. However, a lower value of δ could trigger unnecessary parent change actions when the network can support the current traffic load, which incurs increased overhead.

Next, we introduce a new routing metric $R^{LB}\left(p_{i}\right)$ to consider load balancing when selecting $P_{i}^{*}$:

(7) $$R^{LB}\left(p_{i}\right)=\underset{RANK(p_{i})}{\underset{︸}{H(p_{i})+1}}+ETX\left(n_{i},p_{i}\right)+\lambda BF\left(p_{i}\right),$$

where λ is a weighting coefficient that controls the effect of $BF(p_{i})$ on the parent selection process. Since we have $0\leq BF(p_{i})\leq 1$, we should have λ>1 for $BF(p_{i})$ to have a notable effect compared to $H(p_{i})$ and ETX factors. The impact of λ is discussed later in Section 5. Then, when a congestion is detected, i.e., Equation (6) is satisfied, a new parent $P_{i}^{*}$ is selected as:

(8) $$P_{i}^{*}=\min_{p_{i}\in Parent\left(n_{i}\right)}\left\{R^{LB}\left(p_{i}\right)\right\}.$$

When congestion occurs at $P_{i}$, it is better not to select $n_{i}$ as a parent by its neighbors since all its traffic is eventually forwarded to $P_{i}$, which is already congested. To address this case, $n_{i}$ updates its $BF(n_{i})$ after each parent selection as:

(9) $$BF\left(n_{i}\right)=\max\left\{BF\left(P_{i}\right)-\Delta ,\frac{Q(n_{i})}{L(n_{i})}\right\},$$

where 0<Δ<1 is a small factor. This way, children nodes can be aware of those congested ancestors located up to $\left(\lceil \frac{1}{\Delta }\rceil -1\right)$ hops and avoid selecting parents directly connected to them.

**Figure 3:**
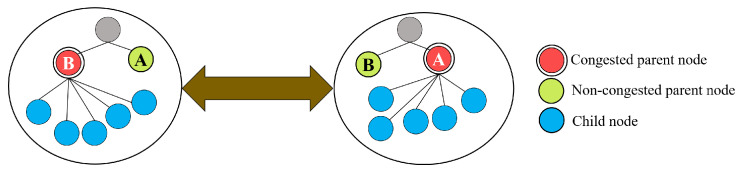
Example of the thundering herd problem.

When utilizing only the LB-criterion to detect the congestion and select a new parent, a number of nodes may simultaneously change to the same parent with the minimum routing metric according to Equation (8). This in turn causes a congestion problem for that new parent. Accordingly, these nodes detect the congestion and change their parent once more, with the minimum backlog factor resulting in congestion again. This may lead to an indefinite cycle of parent changes without achieving a balanced network, a phenomenon that is known as the thundering herd problem [34]. This problem can be illustrated by the scenario shown in Figure 3. To the left, node *B* is congested since it is attached to many children nodes. According to Equations (7) and (8), the children nodes handle the congestion event by changing to a new non-congested parent. The problem is that all the children nodes may simultaneously change to the same new parent, i.e., node *A* as shown in the right side of the figure, which again results in a congestion in that node. In that case, those nodes may continue to change their parent indefinitely without achieving load balancing.

To evade this problem, we introduce a probability-based parent switching mechanism. When the LB-criterion in Equation (6) is satisfied, a node chooses to change to a new parent according to Equation (8) with the following probability:

(10) $$P_{switch}=\max\left\{\Gamma \left(BF\left(P_{i}\right)-BF\left(P_{i}^{*}\right)\right),0\right\},$$

where 0<Γ<1 is a small coefficient that represents the node combativeness to change its parent to avoid congestion. The effect of Γ on the network performance is evaluated in Section 5.

When both the HL-criterion and the LB-criterion are satisfied, the selection mechanism in Equation (8) is applied, because in this case, balancing the network load is more important, as mentioned earlier.

### 3.2. Exchanging the Queue Backlog Information

Neighbor nodes need to share their queue backlog information, i.e., $BF(n_{i})$, in order to be aware of the congestion status. To do so in the method we propose, the value of $BF(n_{i})$ is implicitly embedded into the RANK field in the DIO message in the RPL standard [6]. Accordingly, we change the definition of the RANK in the DIO message to:

(11) $$RANK_{new}\left(n_{i}\right)=\eta \left(H(n_{i})+1\right)+\left(\eta -1\right)BF\left(n_{i}\right),$$

where η is a decoding factor to decode the value of $BF(n_{i})$ (single value) from $RANK_{new}$ (two values). η can be any positive integer value that keeps $RANK_{new}$ within its 16 bit boundary [6]. When a neighbor node receives the DIO message of $n_{i}$, the values of $BF(n_{i})$ and $H(n_{i})$ are decoded separately as follows:

(12) $$\begin{array}{cc} BF(n_{i}) & =\frac{mod\left(RANK_{new}\left(n_{i}\right),\eta \right)}{\eta -1},\\ H(n_{i}) & =\lfloor \frac{RANK_{new}\left(n_{i}\right)}{\eta }\rfloor -1, \end{array}$$

where mod () is the modulo operation. This way, the queue backlog information is distributed among neighbor nodes without the need to change the DIO message format, which ensures that the proposed scheme is compliant with the standard RPL.

### 3.3. Modified Trickle Timer Algorithm

The backlog information should be distributed through the DIO message in a timely manner in order to detect and react quickly to congestion. As mentioned in Section 1, the standard RPL uses the Trickle timer to control the transmission interval of DIO messages. As long as the network is consistent, the DIO message interval is doubled up to a certain maximum value. The timer is reset to a minimum value when inconsistency is detected [10]. However, when the network is consistent, the long DIO interval may cause the nodes to have inaccurate and outdated congestion information, hence fail to achieve load balancing.

**Figure 4:**
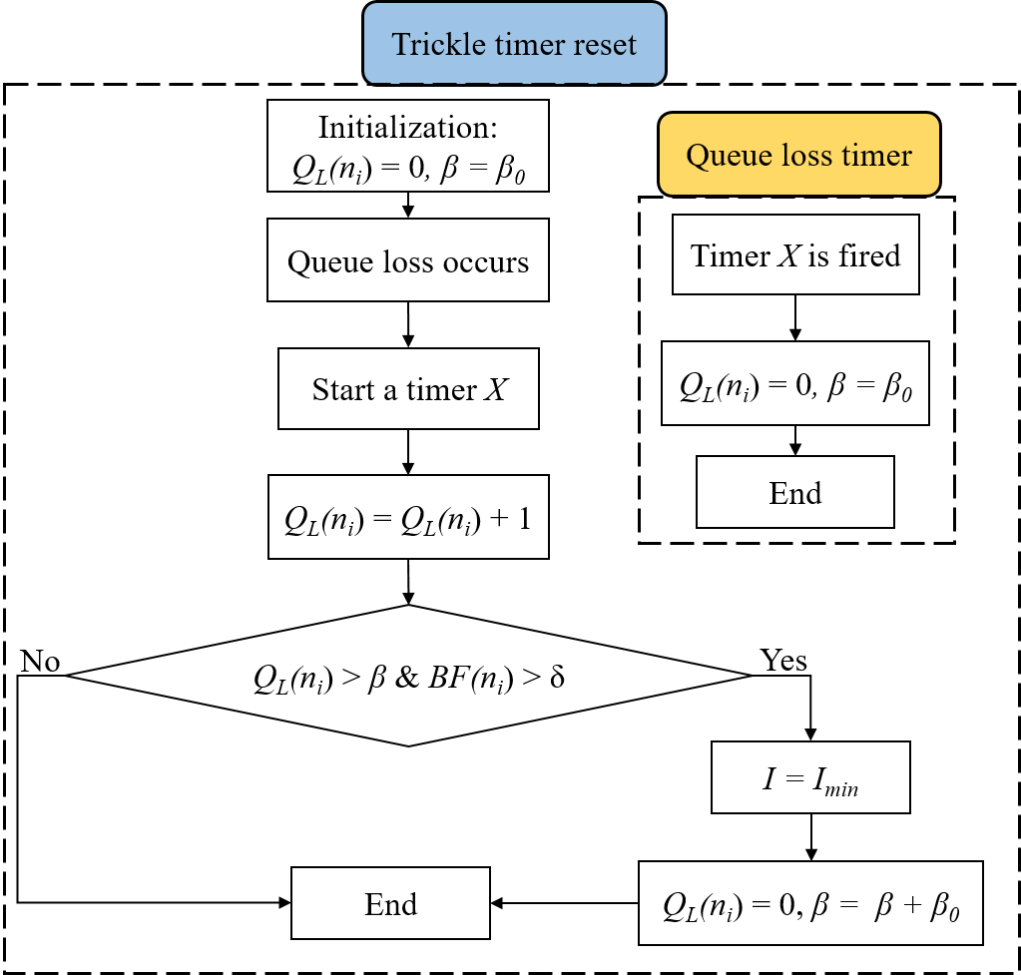
Modified Trickle timer algorithm.

To address this issue, we propose a modification to the Trickle timer in order to distribute the backlog information in a timely manner while keeping the control message overhead to a minimum. The flowchart of the modified Trickle timer algorithm is shown in Figure 4 and described as follows. The basic idea is to reset the Trickle timer interval when the node suffers a certain number of consecutive queue losses $Q_{L}\left(n_{i}\right)$. The intention behind using $Q_{L}\left(n_{i}\right)$ is that LLN nodes have small queues that may fill up temporarily even when there is no congestion in the network. This temporary situation may trigger a false congestion that leads to unnecessary overhead, which can be avoided if we reset the Trickle timer after detecting a particular number of consecutive queue losses. The Trickle timer is reset to its minimum interval $I_{min}$ [10] when $BF(n_{i})$ exceeds δ and $Q_{L}\left(n_{i}\right)$ exceeds a certain limit β. Once a queue loss occurs, a timer *X* is triggered. It is used as a timeout period, that is if no queue losses are detected within this period, the parameters $Q_{L}\left(n_{i}\right)$ and β are reinitialized. When the Trickle timer is reset to $I_{min}$, the value of β is increased by a minimum value $\beta_{0}$ to decrease strictly the number of times the Trickle timer is reset, i.e., minimize the overhead. Therefore, the proposed reset strategy allows the nodes to acquire the queue backlog information through the DIO messages in a timely fashion to act hastily to congestion events, while keeping the DIO messages overhead to a minimum.

The proposed modification in Trickle timer adds insignificant computational complexity. In terms of memory resources, each node needs to store $Q_{L}\left(n_{i}\right)$, $BF(n_{i})$, and β, whose values are not too demanding for storage. In terms of the number of elementary operations, a single sum operation is added, which is executed upon Trickle timer reset, a single increment operation that is executed upon packet loss, and a single subtract operation that is executed when the timer *X* is fired. That is, the computational complexity of the modified reset strategy is O(1).

## 4. Multi-Queue Model and Priority-Based Transmission

In this section, we introduce a priority-based multi-queue transmission model in order to support heterogeneous traffic in 6TiSCH networks. Then, we derive a mathematical model to formulate the average waiting time in each queue.

### 4.1. The Multi-Queue Transmission Model

As discussed in Section 2, the conventional single queue model in 6TiSCH networks cannot guarantee the real-time requirements of high priority data in IIoT applications. To deal with this issue, we design a multi-queue model to support traffic differentiation in heterogeneous 6TiSCH networks in IIoT applications.

Each node exploits a different output queue for each traffic type. We consider a 6TiSCH network that supports up to three traffic types:

- T_1_: represents the safety-critical traffic that has the highest priority, e.g., fire alarms and emergency shutdown.
- T_2_: denotes the acyclic control traffic, which is often time critical. T_2_ has lower priority than T_1_, but higher priority than T_3_.
- T_3_: represents periodic monitoring traffic that is less critical and generated at predictable time instants, e.g., periodic temperature measurements. T_3_ has the lowest priority with relaxed timing requirements.

In our proposed multi-queue model, each node maintains three equally-sized queues, Q_1_, Q_2_, and Q_3_ for T_1_, T_2_, and T_3_, respectively. The packets in Q_1_ are given the highest priority and are always transmitted first, followed by the packets in Q_2_, and lastly, Q_3_, which is given the lowest priority.

In order to elaborate our proposed multi-queue model, we consider an industrial real-world scenario of heterogeneous traffic, which is the process monitoring of plastic extrusion [35]. Plastic extrusion is a high volume manufacturing process in which raw plastic material is melted and formed into a continuous profile to form product items such as pipe/tubing, weather stripping, window frames, adhesive tape, and wire insulation. Firstly, it is critically important to measure pressure in the extruder to prevent serious accidents that can happen when excessively high pressures are generated [36]. Exceeding the safety pressure threshold may ultimately cause an explosion, the barrel may crack, or the die may be blown from the extruder. Melt temperature is also one of the most important variables that has to be maintained very carefully to produce a good quality product. It is important to ensure that the melt is not degraded or overheated during extrusion. A tight temperature profile along the barrel is very important for obtaining a constant quality of plastic material [36]. The whole process could be controlled remotely where all the collected information is transmitted to the Internet through the DODAG root. To map the different traffic within the plastic extrusion scenario to our defined multi-queue model, we have: T_1_ refers to the safety alarms that are generated when the extruder pressure exceeds the predefined threshold to either inform the operator to shut-down the extruder or initiate an automatic shut-down command. T_2_ refers to the control traffic generated when a notable deviation is detected in the temperature profile readings, and this in turn initiates a temperature control mechanism to avoid excessive economical loss. Lastly, T_3_ represents the periodic temperature measurements from the thermocouple temperature sensors.

**Figure 5:**
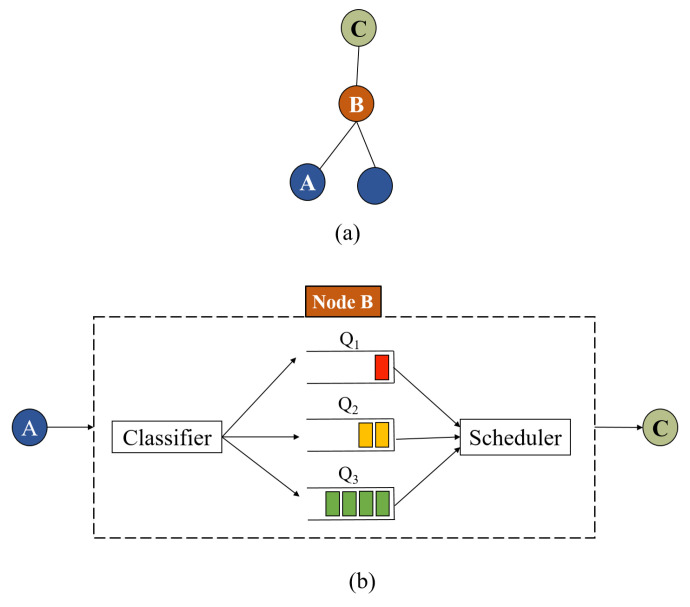
The proposed multi-queue model: (**a**) sub-tree of the Destination-Oriented Acyclic Graph (DODAG); (**b**) multi-queue model of node *B*.

The proposed queuing model is shown in Figure 5, and its working principle is described as follows. A classifier is responsible of sorting the incoming heterogeneous traffic either generated from the node itself or incoming from its children. Based on type and priority, i.e., T_1_, T_2_, or T_3_, the packet is placed in the corresponding queue. The scheduler selects the packet to be transmitted first according to the priority of each queue. If there are T_1_ packets in Q_1_, the scheduler selects the packet to be transmitted first according to the Earliest-Deadline-First (EDF) approach [37], i.e., the T_1_ packet with the minimum absolute deadline is selected. The absolute deadline $D_{i}$ of an arbitrary packet Pk_i_ equals to its arrival time $t_{i}$ plus the relative deadline $d_{i}$, i.e., $D_{i}=t_{i}+d_{i}$. In this context, the relative deadline of a packet is assigned according to the considered application and the information carried in the corresponding packet, e.g., fire alarm, excessive pressure in a pipe, leakage of gas, etc. If Q_1_ is empty, the scheduler selects a packet from Q_2_ to be transmitted first according to the EDF approach. If both Q_1_ and Q_2_ are empty, the packets from Q_3_ are transmitted according to the FIFO policy. Therefore, the packet delay and queuing time are mainly influenced by the number of higher priority packets in the system, as will be illustrated in the next section. The packet category can be embedded in the packet header as a 2 bit field, i.e., 00, 01, and 10 for T_1_, T_2_, and T_3_, respectively, which can be extracted and decoded by the classifier to place the packet in its corresponding queue.

This way, the proposed multi-queue model always ensures a prioritized transmission for the critical data over the non-critical compared to the conventional single-queue model. Another important advantage of the proposed multi-queue scheme is that higher priority data avoid the congestion problem. Traffic types T_1_ and T_2_ occur occasionally; hence, Q_1_ and Q_2_ are likely less prone to congestion problems at parent nodes, i.e., queue overflow and packets loss. Further, this guarantees reliable and real-time communications of critical data even under the heavy traffic load of T_3_.

### 4.2. Mathematical Analysis

In the following, we present a queuing analysis of the proposed multi-queue model where we mathematically formulate the average queue waiting time $\bar{W_{Q_{i}}}$. The formulated $\bar{W_{Q_{i}}}$ corresponds to an arbitrary packet in Q_i_, which will be later referred as the tagged packet. The average queue waiting time of a packet is defined as the time elapsed from the instant the packet is received by the node until the instant it leaves the queue. Each queue in the proposed multi-queue model is represented by a finite-capacity queuing system with a finite storage of *K* [31]. The arrival rate to each queue is modeled based on the generation nature of the corresponding traffic. Since T_1_ and T_2_ are acyclic in nature, we model the packet arrivals to Q_1_ and Q_2_ as a Poisson process with parameters $\alpha_{1}$ and $\alpha_{2}$, respectively. T_3_ traffic is cyclic; thus, packets arrive at Q_3_ periodically with a rate $\alpha_{3}$. In the TSCH schedule, the service time for all packets in each queue is deterministic and equals $T_{c}$ [38], where $T_{c}$ is the horizontal length of the TSCH schedule, i.e., the number of time slots per channel. Therefore, Q_1_ and Q_2_ represent an M/D/1/Kqueuing system, while Q_3_ represents a D/D/1/Kqueuing system [39].

According to our multi-priority model, packets from Q_1_ are always transmitted first following the EDF scheduling policy, i.e., the packet with the shortest deadline will be transmitted first. We consider the general case that the packets in Q_1_ and Q_2_ may join the queue with different deadlines. For instance, considering two packets of the T_1_ type in oil and gas industries, a packet that corresponds to a fire alarm may have a deadline that is different from that of a packet generated to alert about excessive pressure in a pipe. However, our proposed analysis can be simplified to a simple case of all packets within the same traffic category having the same deadline. The basic idea in the following mathematical formulations is to estimate the average number of higher priority packets with respect to an arbitrary tagged packet that would be transmitted ahead of it.

#### 4.2.1. Average Queue Waiting Time in Q_1_

Based on the aforementioned considerations, since the transmission priority of a T_1_ packet within Q_1_ is determined based on the absolute deadline, out of the packets already in Q_1_, there are $N_{1}^{B}$ packets whose absolute deadlines are earlier than that of the tagged packet and scheduled for transmission before it.

**Figure 6:**
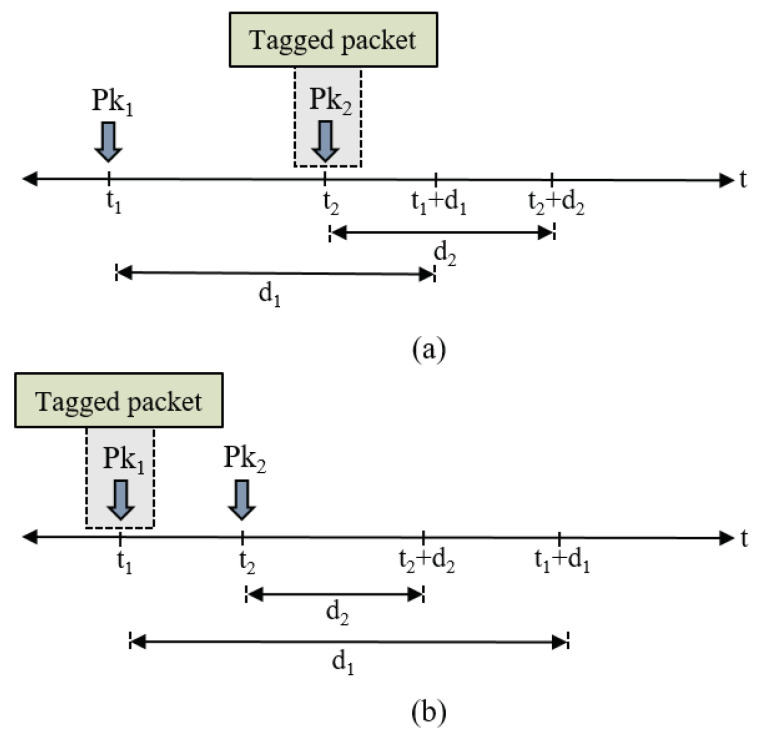
Packet arrivals in the proposed multi-queue model: (**a**) scenario of $N_{1}^{B}$; (**b**) scenario of $N_{1}^{A}$.

This scenario is illustrated by Figure 6a, where we have a packet Pk_1_ with a relative deadline $d_{1}$ and our tagged packet Pk_2_ with a relative deadline $d_{2}$. According to the EDF policy, the transmission priority of each packet within Q_1_ is assigned upon its arrival based on its absolute deadline ($t+d_{i}$). Although $d_{2}<d_{1}$, Pk_1_ has a higher transmission priority than Pk_2_, since the former has an earlier absolute deadline. This applies to the packets arriving at least ($d_{1}-d_{2}$) before the arrival of Pk_2_ and has been waiting for at least ($d_{1}-d_{2}$) given that $(d_{1}-d_{2})>0$. Therefore, we have:

(13) $$\bar{N_{1}^{B}}=\max\left\{0,\bar{\alpha_{1}}\left(\bar{W_{Q_{1}}}-D_{Q_{1}}\right)\right\},$$

where $\bar{\alpha_{1}}$ is the effective arrival rate (we will derive the formula of $\bar{\alpha_{1}}$ later in this section), and $D_{Q_{1}}=d_{1}-d_{2}$, which can be deterministic for constant values of $d_{1}$ and $d_{2}$.

In addition, there are a number of $N_{1}^{A}$ packets that arrive after the tagged packet with an earlier absolute deadline, hence are transmitted first. This case is depicted in Figure 6b, where packet Pk_2_ arrives after the tagged packet Pk_1_, with an earlier absolute deadline. This applies to all packets that arrive after the tagged packet no later than $D_{Q_{1}}$. However, the tagged packet may stay in Q_1_ for a period less than $D_{Q_{1}}$, given that $\bar{W_{Q_{1}}}<D_{Q_{1}}$. Thus, $\bar{N_{1}^{A}}$ is given as:

(14) $$\bar{N_{1}^{A}}=\bar{\alpha_{1}}\min\left\{\bar{W_{Q_{1}}},D_{Q_{1}}\right\}.$$

When those ($N_{1}^{B}+N_{1}^{A}$) packets are transmitted and the tagged packet becomes the one with the highest priority in Q_1_, it will wait for additional time until the beginning of its assigned slot, which is on average $\frac{1}{2}T_{c}$ [38]. Therefore, $\bar{W_{Q_{1}}}$ can be calculated as:

(15) $$\begin{array}{cc} \bar{W_{Q_{1}}} & =\left(T_{c}+\frac{1}{2}T_{c}\right)\left(\bar{N_{1}^{B}}+\bar{N_{1}^{A}}\right)+\frac{1}{2}T_{c}\\ \; & =\frac{T_{c}}{2}\left(3\left(\bar{N_{1}^{B}}+\bar{N_{1}^{A}}\right)+1\right). \end{array}$$

**Figure 7:**
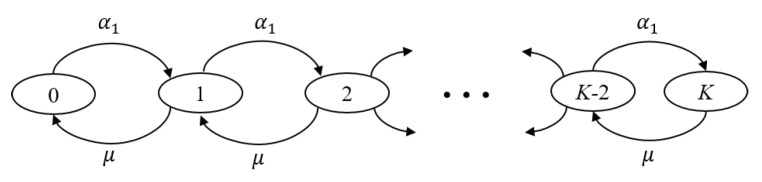
The state-transition diagram for the finite Markov chain of Q_1_.

The next step is to estimate the value of $\bar{\alpha_{1}}$. Since Q_1_ can hold at most *K* packets, T_1_’s packets will continue to be generated according to a Poisson process with parameter $\alpha_{1}$; however, only the packets that find Q_1_ with strictly less than *K* packets will be allowed to join. This is illustrated by Figure 7, which depicts the state-transition diagram for the finite Markov chain of Q_1_, where $\mu =(1/T_{c})$ denotes the service rate. The system can be modeled as a birth-death process [39] where the Poisson input is turned off as soon as Q_1_ is filled up. Therefore, we have:

(16) $$\alpha_{1k}=\left\{\begin{array}{cc} \alpha_{1} & k<K\\ 0 & k\geq K, \end{array}\right.$$

where $\alpha_{1k}$ is the birth rate at Q_1_ when it includes *k* packets. It is worth mentioning that the system in Figure 7 and the following derivations are also valid for Q_2_ and Q_3_. Using Equation (16), the effective arrival rate $\bar{\alpha_{1}}$ can be given as:

(17) $$\bar{\alpha_{1}}=\sum_{k=0}^{K}\alpha_{1k}\;p_{k}=\sum_{k=0}^{K-1}\alpha_{1}p_{k},$$

where $p_{k}$ is the steady-state probability to find *k* packets in Q_1_. Solving the equilibrium equations for the queuing system in Figure 7, we obtain:

(18) $$p_{k}=\left\{\begin{array}{cc} p_{0}{\left(\frac{\alpha_{1}}{\mu }\right)}^{k} & 0\leq k\leq K\\ 0 & k>K. \end{array}\right.$$

Using Equation (18) along with the conservation relation $\sum_{k=0}^{\infty }p_{k}=1$, we solve for $p_{0}$:

(19) $$\begin{array}{cc} p_{0} & =\frac{1}{1+\sum_{k=1}^{K}{\left(\frac{\alpha_{1}}{\mu }\right)}^{k}}={\left[1+\frac{\left(\frac{\alpha_{1}}{\mu }\right)\left(1-{\left(\frac{\alpha_{1}}{\mu }\right)}^{K}\right)}{1-\frac{\alpha_{1}}{\mu }}\right]}^{-1}\\ \; & =\frac{1-\frac{\alpha_{1}}{\mu }}{1-{\left(\frac{\alpha_{1}}{\mu }\right)}^{\left(K+1\right)}}. \end{array}$$

Then, $p_{k}$ in Equation (18) can be rewritten as:

(20) $$p_{k}=\left\{\begin{array}{cc} \frac{{\left(\frac{\alpha_{1}}{\mu }\right)}^{k}\left(1-\frac{\alpha_{1}}{\mu }\right)}{1-{\left(\frac{\alpha_{1}}{\mu }\right)}^{\left(K+1\right)}} & 0\leq k\leq K\\ 0 & k>K. \end{array}\right.$$

Applying Equation (20) in Equation (17), we get:

(21) $$\begin{array}{cc} \bar{\alpha_{1}} & =\alpha_{1}\sum_{k=0}^{K}\frac{{\left(\frac{\alpha_{1}}{\mu }\right)}^{k}\left(1-\frac{\alpha_{1}}{\mu }\right)}{1-{\left(\frac{\alpha_{1}}{\mu }\right)}^{\left(K+1\right)}}\\ \; & =\alpha_{1}\frac{1-{\left(\frac{\alpha_{1}}{\mu }\right)}^{K}}{1-{\left(\frac{\alpha_{1}}{\mu }\right)}^{\left(K+1\right)}}\\ \; & =\alpha_{1}\frac{1-{\left(\alpha_{1}T_{c}\right)}^{K}}{1-{\left(\alpha_{1}T_{c}\right)}^{\left(K+1\right)}}. \end{array}$$

Based on Equations (13)–(15), and (21), $\bar{W_{Q_{1}}}$ can be given as:

(22) $$\bar{W_{Q_{1}}}=\frac{T_{c}}{2}\left(3\left(\left(\alpha_{1}\frac{1-{\left(\alpha_{1}T_{c}\right)}^{K}}{1-{\left(\alpha_{1}T_{c}\right)}^{\left(K+1\right)}}\right)\left(\max\left\{0,\left(\bar{W_{Q_{1}}}-D_{Q_{1}}\right)\right\}+\min\left\{\bar{W_{Q_{1}}},D_{Q_{1}}\right\}\right)\right)+1\right).$$

#### 4.2.2. Average Queue Waiting Time in Q_2_

The average queue waiting time $\bar{W_{Q_{2}}}$ of a tagged T_2_ packet depends not only on the packets found in Q_1_ upon arrival, but also the subsequent arrivals of T_1_ packets. According to Little’s formula [39], there are on average $\bar{\alpha_{1}}\bar{W_{Q_{1}}}$ packets found in Q_1_; in addition, a number of T_1_ packets may arrive while the tagged packet waits in Q_2_, which is on average $\bar{\alpha_{1}}\bar{W_{Q_{2}}}$ packets. Since Q_2_ follows the EDF approach to schedule T_2_ packets, it follows the same scenario illustrated in Figure 6. Hence, $\bar{W_{Q_{2}}}$ can be expressed as follows:

(23) $$\bar{W_{Q_{2}}}=T_{c}\left(\frac{3}{2}\left(\bar{\alpha_{1}}\bar{W_{Q_{1}}}+\bar{\alpha_{1}}\bar{W_{Q_{2}}}+\bar{N_{2}^{B}}+\bar{N_{2}^{A}}\right)+\frac{1}{2}\right).$$

Following the same procedures of Equations (13)–(21), $\bar{W_{Q_{2}}}$ is given as:

(24) $$\begin{array}{cc} \bar{W_{Q_{2}}} & =\frac{T_{c}}{2}\left(3\left(\left(\alpha_{1}\frac{1-{\left(\alpha_{1}T_{c}\right)}^{K}}{1-{\left(\alpha_{1}T_{c}\right)}^{\left(K+1\right)}}\right)\left(\bar{W_{Q_{1}}}+\bar{W_{Q_{2}}}\right)\right.\right.\\ \; & +\left.\left.\left(\alpha_{2}\frac{1-{\left(\alpha_{2}T_{c}\right)}^{K}}{1-{\left(\alpha_{2}T_{c}\right)}^{\left(K+1\right)}}\right)\left(\max\left\{0,\left(\bar{W_{Q_{2}}}-D_{Q_{2}}\right)\right\}+\min\left\{\bar{W_{Q_{2}}},D_{Q_{2}}\right\}\right)\right)+1\right). \end{array}$$

#### 4.2.3. Average Queue Waiting Time in Q_3_

According to the FIFO approach in Q_3_, an arbitrary tagged T_3_ packet has to wait for the transmission of all packets that arrived ahead. Since Q_3_ has the lowest priority, $\bar{W_{Q_{3}}}$ is directly affected by the arrivals of T_1_ and T_2_. In addition to the average number of packets already waiting in Q_1_, Q_2_, and Q_3_, which are $\bar{\alpha_{1}}\bar{W_{Q_{1}}}$, $\bar{\alpha_{2}}\bar{W_{Q_{2}}}$, and $\bar{\alpha_{3}}\bar{W_{Q_{3}}}$, respectively, a tagged packet that arrives at Q_3_ has to wait for higher priority packets to arrive at Q_1_ and Q_2_, which are on average $\bar{\alpha_{1}}\bar{W_{Q_{3}}}+\bar{\alpha_{2}}\bar{W_{Q_{3}}}$ packets. Accordingly, $\bar{W_{Q_{3}}}$ is given as:

(25) $$\begin{array}{cc} \bar{W_{Q_{3}}} & =\frac{T_{c}}{2}\left(3\left(\bar{\alpha_{1}}\bar{W_{Q_{1}}}+\bar{\alpha_{2}}\bar{W_{Q_{2}}}+\bar{\alpha_{3}}\bar{W_{Q_{3}}}+\bar{\alpha_{1}}\bar{W_{Q_{3}}}+\bar{\alpha_{2}}\bar{W_{Q_{3}}}\right)+1\right)\\ \; & =\frac{T_{c}}{2}\left(3\left(\left(\alpha_{1}\frac{1-{\left(\alpha_{1}T_{c}\right)}^{K}}{1-{\left(\alpha_{1}T_{c}\right)}^{\left(K+1\right)}}\right)\left(\bar{W_{Q_{1}}}+\bar{W_{Q_{3}}}\right)+\left(\alpha_{2}\frac{1-{\left(\alpha_{2}T_{c}\right)}^{K}}{1-{\left(\alpha_{2}T_{c}\right)}^{\left(K+1\right)}}\right)\left(\bar{W_{Q_{2}}}+\bar{W_{Q_{3}}}\right)\right.\right.\\ \; & +\left.\left.\left(\alpha_{3}\frac{1-{\left(\alpha_{3}T_{c}\right)}^{K}}{1-{\left(\alpha_{3}T_{c}\right)}^{\left(K+1\right)}}\right)\bar{W_{Q_{3}}}\right)+1\right). \end{array}$$

The average queue waiting times given in Equations (22), (24), and (25) must satisfy the Kleinrock conservation law [40]:

(26) $$\sum_{i=1}^{3}\rho_{i}\bar{W_{Q_{i}}}=\frac{\sum_{i=1}^{3}\rho_{i}\bar{W_{0}}}{1-\sum_{i=1}^{3}\rho_{i}},$$

where $\rho_{i}=\bar{S_{i}}\bar{\alpha_{i}}$ is the utilization factor of Q_i_, $\bar{S_{i}}$ is the average service time of packets in Q_i_, and $\bar{W_{0}}$ is the average residual service time of the packet whose transmission is in progress. According to the mean residual life formula [40], $\bar{W_{0}}$ is given as:

(27) $$\bar{W_{0}}=\sum_{i=1}^{3}\rho_{i}\frac{\bar{{S_{i}}^{2}}}{2\bar{S_{i}}}=\frac{{T_{c}}^{2}}{2}\sum_{i=1}^{3}\bar{\alpha_{i}}.$$

Since we have a deterministic service time for Q_1_, Q_2_ and Q_3_, i.e., $\bar{{S_{i}}^{2}}={T_{c}}^{2}$, $\bar{W_{0}}$ in Equation (27) can be obtained as follows:

(28) $$\bar{W_{0}}=\frac{{T_{c}}^{2}}{2}\left(\alpha_{1}\frac{1-{\left(\alpha_{1}T_{c}\right)}^{K}}{1-{\left(\alpha_{1}T_{c}\right)}^{\left(K+1\right)}}+\alpha_{2}\frac{1-{\left(\alpha_{2}T_{c}\right)}^{K}}{1-{\left(\alpha_{2}T_{c}\right)}^{\left(K+1\right)}}+\alpha_{3}\frac{1-{\left(\alpha_{3}T_{c}\right)}^{K}}{1-{\left(\alpha_{3}T_{c}\right)}^{\left(K+1\right)}}\right)$$

Having deterministic values for $T_{c},D_{Q_{1}}$, and $D_{Q_{2}}$, the average queue waiting time $\bar{W_{Q_{i}}}$ of each queue can be solved via the set of non-linear Equations (22), (24), and (25) along with the conservation formula given by Equation (26).

## 5. Performance Evaluations

We evaluated the performance of our proposed work and compared it with existing work under different performance metrics. The results were obtained through extensive Monte Carlo simulations in MATLAB. We used the parameters defined in Table 1 to model the simulation environment as close to the real conditions as possible.

We considered a network of 30 nodes that were randomly deployed in a 200 m × 200 m area. Communications were carried out through a predefined TSCH schedule. Constructing and maintaining the TSCH schedule were out of scope of this paper. We considered log-normal shadowing distribution for the channel model with the specified standard deviation selected according to the measurements reported in [41]. Each of the following results were averaged over 10 simulation runs with each lasting for a duration of 1000 consecutive slotframes. The results were produced with a 95% confidence interval based on the t-distribution. In the following text and figures, we refer to our proposed Congestion Control and Traffic Differentiation method as CCTD. We evaluated the performance of CCTD under two scenarios. The first scenario considered a single-traffic network model where nodes generated only T_3_ traffic periodically according to a specific rate. The second scenario considered a multi-traffic network model where each node generated both T_1_ and T_2_ packets according to a Poisson process with parameters $\alpha_{1}$ and $\alpha_{2}$, respectively, as defined in Table 1, along with the periodic T_3_ packets. For both scenarios, we considered a fixed packet size of 100 for all traffic types [42]. Since timeliness and reliability were of primary importance to support IIoT applications, our results mainly focused on the Packet Delivery Ratio (PDR) and End-to-End (E2E) delay parameters.

### 5.1. Single-Traffic Scenario

**Figure 8:**
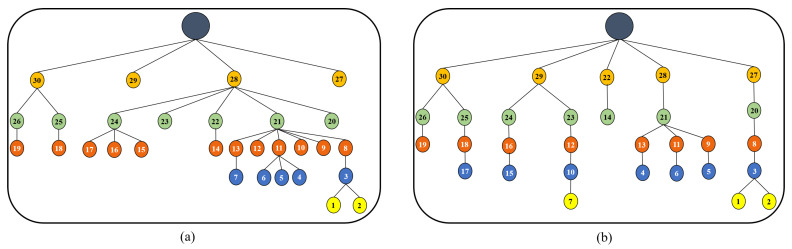
DODAG created by: (**a**) RPL-Objective Function (OF) 0; (**b**) Congestion Control and Traffic Differentiation (CCTD).

First, we describe the effect of the proposed congestion control method on the network topology by sketching the DODAG created by RPL-OF0 [8] and CCTD in Figure 8a,b, respectively. Each DODAG structure in Figure 8 represents the routing topology recognized at the end of the simulations by tracking each child-parent pair in each end-to-end path through the node ID. As shown in the figure, the DODAG created by RPL-OF0 suffered from imbalanced load distribution among parent nodes. For instance, Node 21 had the burden to forward almost 43% of the total load of the network through its corresponding sub-tree, which was much less than other nodes with the same RANK, e.g., Nodes 20, 23, and 25. The situation was even worse for Node 28, which had the responsibility to forward the traffic of almost 70% of the network. This imbalanced network was mainly due to the adopted parent selection mechanism in RPL-OF0, which was unaware of the congestion situation at parent nodes, and hence imposed significant degradation in the network performance in terms of delay and packet delivery. However, the proposed CCTD distributed the traffic load fairly among intermediate nodes, as shown in Figure 8b. The CCTD approach managed to reduce the standard deviation of the number of children per node from 1.6 to 0.73. This was due to the adopted congestion-aware parent selection mechanism in CCTD where a node selected its preferred parent according to its queue occupancy, which was updated efficiently through the improved Trickle timer algorithm.

**Figure 9:**
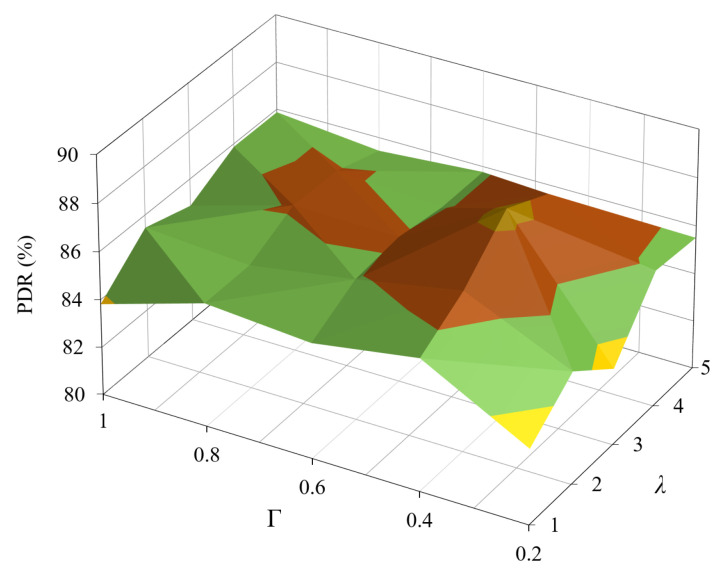
PDR performance under different values of λ and Γ at a traffic load of 90 ppm/node.

Next, we evaluated the impact of the design parameters λ and Γ on the performance of the proposed CCTD. Figure 9 shows the PDR at a packet generation rate of 90 packets per minute (ppm). The PDR was calculated by dividing the number of packets successfully received at the DODAG root by the total packets generated in the network. We first observed that the PDR improved as λ increased due to the fact that increasing λ in Equation (7) made the parent switching mechanism mainly based on the queue backlog factor to avoid congestion, hence improving the PDR. However, the PDR then decreased until it stayed almost constant as larger values of λ may cause the node to select parents with longer paths and/or unreliable links. Furthermore, we observed that the PDR performance almost followed the same trend with varying Γ. For large values of Γ, the nodes had a high tendency to change their parents as soon as a congestion was detected, which constituted the trade-off between fast load balance and the thundering herd effect as mentioned in Section 3. Therefore, the values of λ and Γ could be empirically adjusted by observing network performance. Based on Figure 9, we selected λ=4 and Γ=0.5 for the following results as these values gave the best PDR performance.

**Figure 10:**
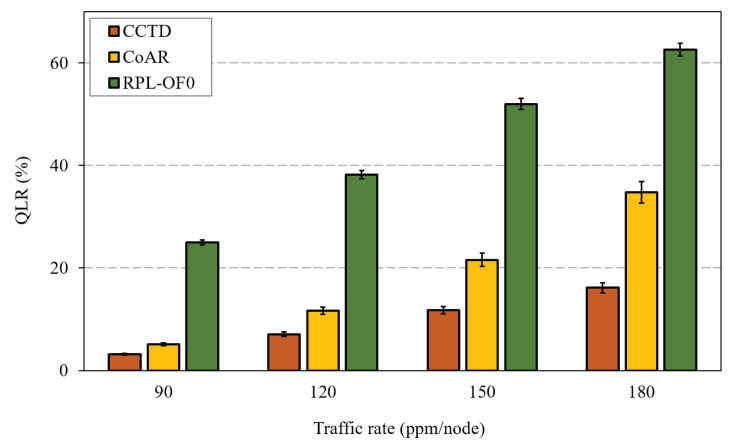
Queue Loss Ratio (QLR) comparison for different traffic rates.

Hereafter, we compare the proposed work with RPL-OF0 and CoAR [30] under different performance metrics. Figure 10 shows the Queue Loss Ratio (QLR) of the three schemes for different traffic rates. The QLR was calculated as the total packets lost due to queue overflow divided by the total generated packets in the network. Although queue losses and buffer overflow are inevitable under heavy load conditions, efficient load-balancing and congestion control could mitigate such problems. The proposed CCTD approach managed to reduce the QLR of RPL-OF0 by 79% at 150 ppm/node as a result of the adopted congestion control framework, which helped achieve a fair load distribution among intermediate nodes. On the other hand, the proposed probabilistic parent selection strategy in Equation (10) and the improved Trickle timer algorithm together helped to improve the QLR performance compared to that achieved by CoAR. For instance, CCTD improved the QLR of CoAR by 45% at 150 ppm/node, which was increased to 53% at 180 ppm/node.

**Figure 11:**
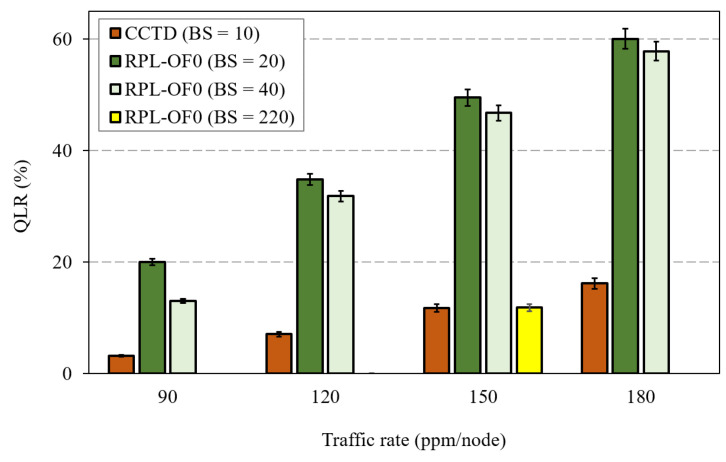
Effect of the buffer size increase on QLR.

As mentioned earlier, RPL was implemented on LLN resource-constrained nodes with small queue sizes, and these queues started to overflow under heavy traffic load. A solution could be to increase the buffer size of intermediate nodes to alleviate the congestion problem. However, such a solution was ineffective in the case of imbalanced RPL networks without a proper parent selection mechanism. To further illustrate, Figure 11 shows the QLR performance of RPL-OF0 after increasing the Buffer Size, denoted as BS in the figure, to 20 and 40. For the sake of comparison, we also added the QLR of CCTD with the BS of 10 packets. As depicted by Figure 11, increasing the buffer size to the double value marginally improved the QLR in RPL-OF0 under heavy traffic conditions. For instance, the QLR was reduced by 9% when increasing the BS from 20 to 40 at 120 ppm/node, while it was reduced to only 3% at a traffic rate of 180 ppm/node. On the other hand, CCTD with a BS of 10 maintained improved QLR performance over RPL-OF0 under heavy traffic rates. In order to further prove the effectiveness of the proposed method, Figure 11 shows that at a rate of 150 ppm/node, RPL-OF0 could achieve almost the same QLR as CCTD when increasing the BS to 220. Such a queue size is, however, infeasible in practice given the current resource-constrained LLN devices.

**Figure 12:**
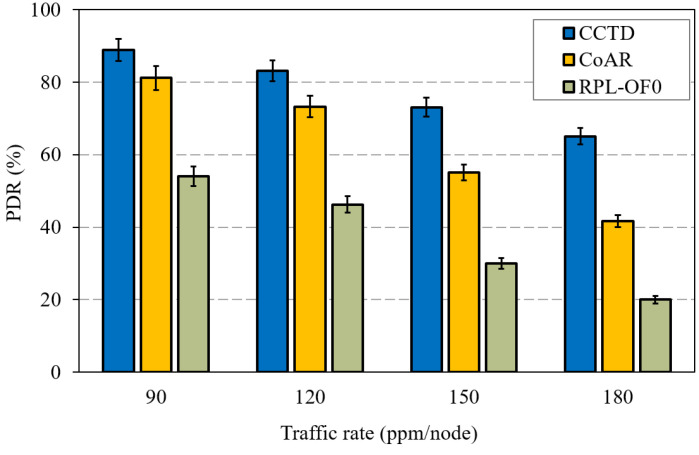
PDR performance comparison for different traffic rates.

As the QLR reduced, the proposed CCTD in turn enhanced the PDR performance compared to RPL-OF0 and CoAR, as shown in Figure 12. The effectiveness of the proposed CCTD was clearly observed at heavy load conditions where CCTD improved the performance of CoAR by 33% and 59% at 150 ppm/node and 180 ppm/node, respectively.

**Figure 13:**
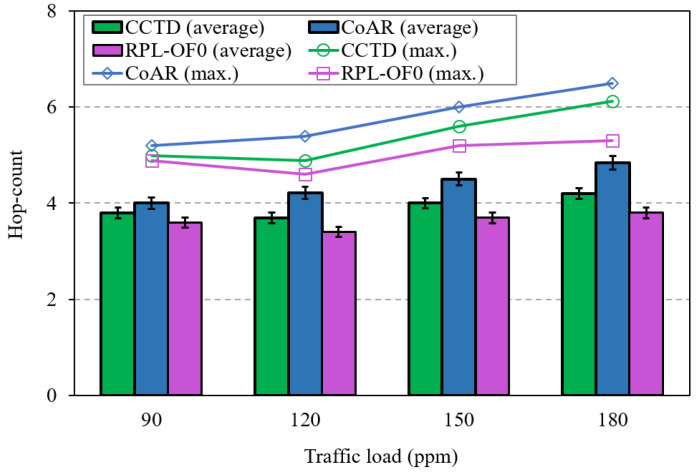
Hop-count comparison for different traffic rates.

Figure 13 shows the hop-count comparison between the three methods against different traffic rates. The figure includes both the average and the maximum hop-count to the DODAG root. As can be noted from the figure, the proposed CCTD had a marginal effect on the hop-count of RPL-OF0 as nodes may select a path with a higher hop distance towards the DODAG root to alleviate the congestion effect. Moreover, CCTD exploited the hop-count metric in $R^{LB}\left(p_{i}\right)$ as shown by (7) when selecting the alternative parent; therefore, CCTD incurred a slight increase in this metric. Furthermore, the parameter λ could be further tuned to achieve a trade-off between congestion control and hop-count. However, since CoAR completely excluded the hop-count from the routing metric in its congestion control mechanism, it showed a higher hop-count than that of CCTD and RPL-OF0.

**Figure 14:**
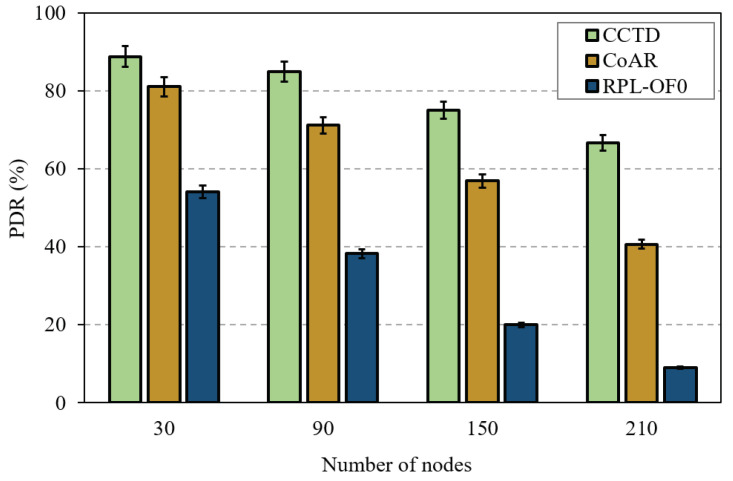
PDR performance comparison for different network sizes with a traffic rate of 90 ppm/node.

For a quick look at the effect of the network size on the performance of the proposed CCTD, Figure 14 shows a PDR comparison of the three schemes with a varying number of nodes in the network. First, we observed that the PDR performance of RPL-OF0 significantly degraded as the network size increased. This was because the network was at a great risk of suffering from an imbalanced load distribution in terms of DODAG construction, which caused severe queue losses. Hence, congestion control and load balancing become more important and an effective way to improve the performance of RPL in large-scale networks. Accordingly, as shown in Figure 14, CCTD maintained its PDR performance enhancement and even achieved more improvements as the network size increases. Specifically, CCTD improved the PDR by 64% compared to RPL-OF0 with 30 nodes, while the percentage was dramatically increased to 275% with 150 nodes. In this context, it is reported that in process automation scenarios, the network would include a maximum of 50 nodes in order to meet the required refresh rates [43].

### 5.2. Multi-Traffic Scenario

Hereafter, we consider the heterogeneous network model to emphasize the effect of the proposed multi-queue model in CCTD.

**Figure 15:**
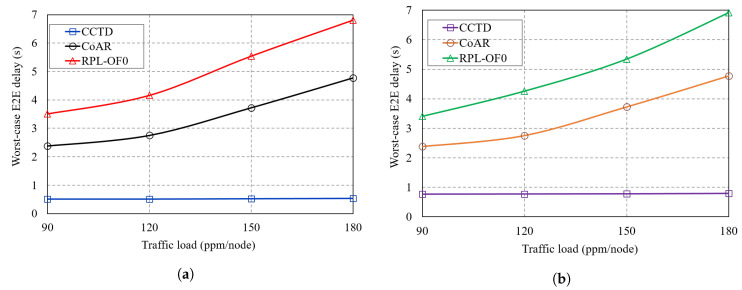
Worst-case delay comparison of: (**a**) T_1_ traffic; (**b**) T_2_ traffic.

Figure 15a,b shows the worst-case E2E comparison of T_1_ and T_2_ traffic, respectively, under different traffic rates of T_3_. The E2E delay was calculated as the time elapsed from the generation of the packet until it was successfully delivered to the DODAG root. As shown in the two figures, the proposed CCTD scheme significantly reduced the worst-case delay of critical traffic T_1_ and T_2_ compared to RPL-OF0 and CoAR. With the proposed priority-based multi-queue model, critical packets were always given higher priority for transmission as long as Q_1_ and Q_2_ were non-empty where packets from Q_1_ were given the highest priority. Such a function was not provided either by RPL-OF0 or CoAR, where packet transmissions followed an FIFO-based single-queue model regardless of the priority and the criticality of the traffic. Hence, critical data were likely to be blocked by the transmission of multiple non-critical data as the latter arrived first. The situation became even worse at high traffic rates of T_3_ as more traffic was queued ahead to the critical data, which further degraded its delay performance, while CCTD preserved a steady lower delay regardless of the T_3_ traffic load as shown in Figure 15a,b. Moreover, buffer overflow was likely to occur in RPL-OF0 and CoAR with the single queue model in heavy traffic scenarios where T_1_ and T_2_ packets were dropped and needed to be retransmitted, which added significantly to the delay. Quantitatively, compared to CoAR, CCTD reduced the worst-case delay of T_1_ by at least 78% while it achieved a 68% reduction for T_2_.

**Figure 16:**
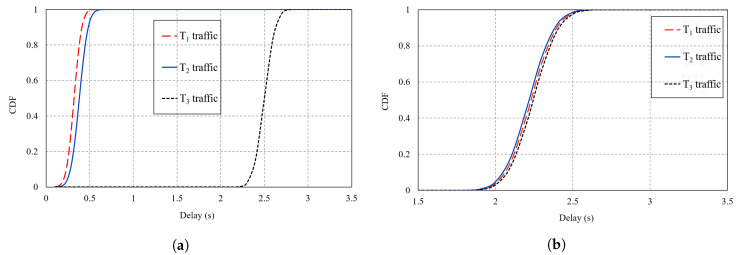
CDF of the E2E delay of all traffic types at 120 ppm/node: (**a**) CCTD; (**b**) Congestion-Aware Routing (CoAR).

The introduced service differentiation was further illustrated through the Cumulative Distribution Function (CDF) comparison of E2E delay as shown by Figure 16a,b where we plot the CDF at 120 ppm/node in CCTD and CoAR, respectively. Figure 16a clearly shows the service differentiation between the different traffic types in CCTD as a result of the proposed multi-queue model where T_1_ packets in Q_1_ were given the highest priority priority, while T_3_ packets were given the lowest priority. However, Figure 16b shows that all traffic in CoAR almost followed the same trend for the E2E delay distribution. In CoAR, the packet in the output buffer followed the FIFO scheduling policy; hence, on average, all packets experienced identical E2E delay.

The results in Figure 16a also reflected on-time PDR of both T_1_ and T_2_. The on-time PDR denotes the percentage of packets delivered within a predefined deadline. As mentioned earlier, for critical traffic, out of the total packets delivered successfully to the DODAG root, those delivered within the specified deadline limit were meaningful to the system. For instance, CCTD achieved a one-time PDR of 86% considering a deadline of 400 ms for T_1_, while the percentage was 92% considering a deadline of 500 ms for T_2_.

**Figure 17:**
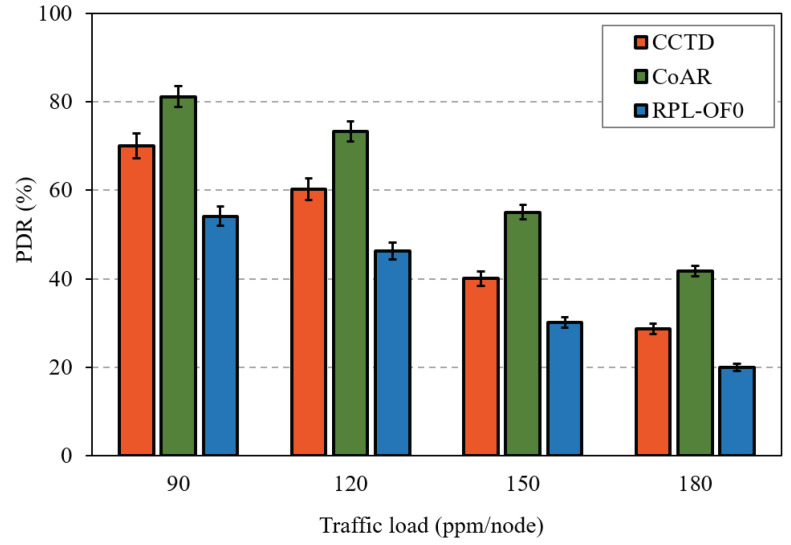
PDR comparison of T_3_ for different traffic rates.

The introduced multi-queue model in CCTD had a direct effect on the delivery performance of T_3_, as the buffered T_3_ packets in Q_3_ were suspended from transmission as long as Q_1_ and/or Q_2_ were non-empty. Hence, CCTD sacrificed the reduced PDR performance of T_3_ in favor of the improved performance for T_1_ and T_2_. Figure 17 shows the PDR comparison of T_3_ for different traffic rates. CCTD had a reduction of 32% in the PDR of T_3_ at 180 ppm/node compared to CoAR, which was relaxed to 13% at 90 ppm/node. However, such degradation could be considered insignificant in critical IIoT applications where guaranteeing real-time performance of critical data is a primary priority [17]. In addition, T_1_ and T_2_ occurred infrequently, and such degradation was not persistent in the network. However, CCTD preserved improved PDR performance against RPL-OF0 due to the proposed congestion control mechanism, which greatly compensated the degradation caused by the multi-queue model.

**Figure 18:**
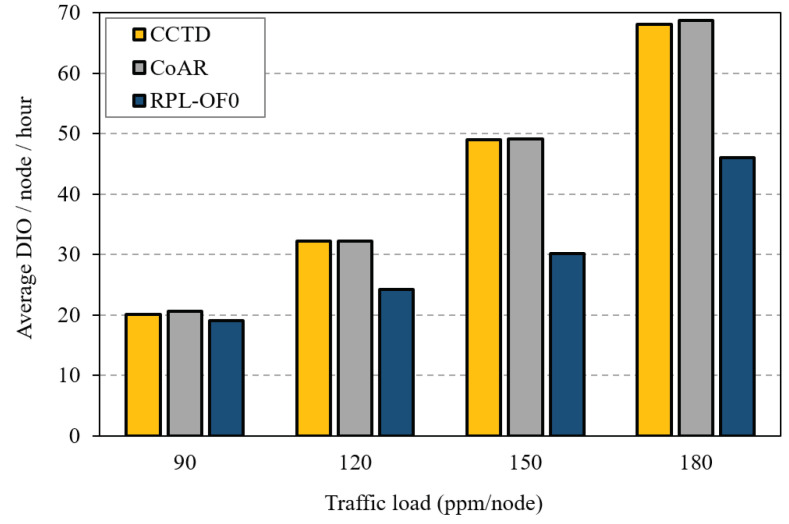
Average number of DODAG Information Object (DIO) messages for different traffic rates of T_3_.

Finally, we investigated the routing overhead introduced by the threes schemes in terms of average number of transmitted DIO messages. In this context, Figure 18 shows the average DIO messages transmitted per node per hour. As shown in the figure, both CCTD and CoAR incurred additional DIO overhead compared to that in RPL-OF0, especially under heavy traffic load. This was because nodes in CCTD and CoAR transmitted more DIO messages to distribute the congestion information based on the corresponding Trickle timer reset strategy in each scheme. The increase in the overhead, however, could be considered a reasonable cost to achieve a fair load distribution in the network and avoid the severe consequences of network congestion. Furthermore, the additional overhead was generally insignificant compared to the total active traffic in the network. For instance, at 90 ppm/node, the overhead in CCTD represented less than 1% of the total traffic.

## 6. Conclusions and Future Work

In this paper, we proposed a congestion control and service differentiation strategy to support heterogeneous 6TiSCH networks in IIoT applications. The congestion was monitored through sharing the backlog information among neighbor nodes, which was implicitly embedded in the DIO message. The preferred parent of each node was selected based on its queue occupancy to achieve a fair load distribution in the network. A modified Trickle timer strategy was also introduced to detect and act on congestion in timely manner while keeping overhead to a minimum. Moreover, we proposed a multi-queue model to support prioritized and low delay transmission of critical traffic. In addition, we provided a mathematical analysis of the average waiting time of each priority queue. Performance evaluations were carried out, and the results proved the effectiveness of our method to achieve improved packet delivery and low queue losses under heavy load scenarios, as well as the improved delay performance of critical traffic with an insignificant increase in the overhead.

As future work, the performance of the proposed framework can be further investigated in terms of power consumption and network lifetime metrics, as well as providing mathematical models for the throughput and worst-case delay of each traffic category. Moreover, we plan to have a full implementation of the proposed framework to evaluate its performance in real scenarios. Optimizing the TSCH schedule to further improve the channel access and transmission delay of critical data is also left as a future investigation.

## Figures and Tables

**Table 1:** Simulation parameters.

Parameter	Value
Network size	30 nodes
Propagation model	Shadowing (log-normal)
Standard deviation	14 dB
Deployment area	200 m × 200 m
Transmission range	30 m
Data rate	250 k/s
Packet length	100 B
Slotframe length	200 slots
Time slot duration	10 ms
No. of channels	4
Output buffer size	10 packets
No. of retransmissions	3
$I_{min}$	3 s
Δ	0.25
θ	0.5
δ	0.5
*m*	4
$\alpha_{1}$	1/50 s
$\alpha_{2}$	1/20 s
